# Multiple sclerosis treatment review for primary care providers

**DOI:** 10.1097/01.NPR.0000000000000202

**Published:** 2024-06-25

**Authors:** Jeffrey Hernandez

**Affiliations:** **Jeffrey Hernandez** is an MS-certified NP and supervisor of advanced practice providers at the MS Center at University of Miami in Miami, Fla.

**Keywords:** disease-modifying therapy, multiple sclerosis, multiple sclerosis treatment, neurologic disorder, neurology

## Abstract

The treatment landscape for multiple sclerosis has dramatically grown in terms of available options and complexity. The various mechanisms of action and safety profiles of these new treatments necessitate that primary care providers remain current in knowledge and practice to provide high-quality care.

**Figure:**
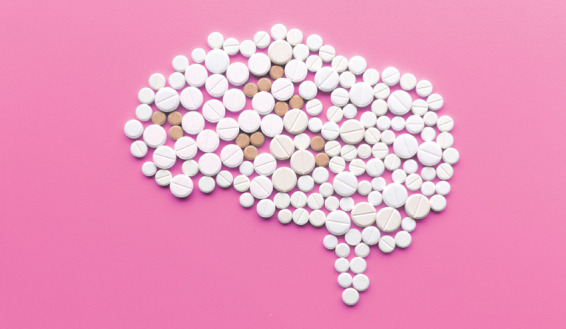
No caption available.

Multiple sclerosis (MS) is the most common neurologic disease of young adults worldwide and impacts nearly 1 million people in the US.^1^ Since 2020, more than five disease-modifying therapies (DMTs) have been added to the armamentarium against MS. The increasing complexity of MS treatment options requires specialists and primary care providers (PCPs) to collaborate more closely to provide high-quality care. Given that many currently approved DMTs work differently and have varying safety profiles, this article aims to provide a brief overview of MS epidemiology, pathophysiology, clinical manifestations, diagnostic workup, and available treatment options.

## EPIDEMIOLOGY

Data suggest that the incidence of MS has increased, and a recent prevalence study revealed that nearly 1 million individuals are living with MS in the US.^1^,^2^ Prevalence varies but is highest in regions furthest from the Equator.^1^-^4^ Although it was long believed that MS was primarily a disease affecting the White population of Northern European descent, more recent studies suggest a higher incidence of MS in the Black population in the US than previously thought. Even across racial groups, MS is three times more common in women than in men.^1^-^3^,^5^

The exact cause of MS is unknown. However, growing data suggest that multiple risk factors contribute to its development.^2^,^3^,^5^ More than 200 independent genetic variants have been identified as associated with an increased risk of MS.^6^ Although no one specific variant is sufficient to cause the disease, human leukocyte antigen (HLA)-DRB1∗15:01, which is present in about 30% of the US and Northern European populations, has been reported as the primary genetic variant associated with increased risk of MS.^3^,^5^,^6^

Additional risk factors for developing MS include obesity during childhood or adolescence, smoking, passive smoking during childhood, lower vitamin D levels, and a history of Epstein-Barr virus (EBV) infection.^2^,^3^,^5^,^7^ EBV, a member of the herpes virus family, is a common cause of asymptomatic infection during childhood or infectious mononucleosis among teenagers and young adults.^3^ A large seroepidemiologic study in Germany reported that 100% (N = 901) of participants with early MS tested positive for EBV antibodies, which was higher than the comparison group, further supporting the role of infection with EBV as a risk factor for MS.^7^

## PATHOPHYSIOLOGY

In MS, patients develop lesions throughout multiple areas of the central nervous system (CNS), specifically the brain, optic nerves, and spinal cord. Most patients experience acute lesions early in the disease due to inflammatory activity by immune cells that cross the CNS through an altered blood-brain barrier, as evidenced by gadolinium-enhancing lesions on MRI.^3^-^5^,^8^,^9^ As a result of the damage, demyelination and neuronal injury occur.^3^-^5^,^8^-^10^

MS was once thought to be a T-cell mediated autoimmune disease, but growing evidence indicates that multiple immune cells play a role in MS relapses and progression.^3^-^5^,^8^-^10^ According to Bar-Or and Li, acute lesions in MS relapses develop due to CNS inflammation caused by autoreactive T cells, B cells, and myeloid cells.^3^,^9^,^10^ Activated proinflammatory microglia, CD4+ T cells, and CD8+ T cells lead to the destruction of myelin and simultaneously serve to recruit other immune cells that further contribute to demyelination and neuronal injury.^3^ CD4+ and CD8+ T cells have long been implicated in the inflammatory process. However, the role of B cells has more recently been recognized in MS as influencing the inflammatory response through antibody production, antigen presentation to T cells, and immunoregulatory cytokine production.^3^,^5^,^8^-^10^ The success of clinical trials in reducing CNS inflammation by depleting B cells with anti-CD20 agents further supports the role of B cells in MS pathophysiology.

## CLINICAL MANIFESTATIONS

The most common subtype of MS is relapsing MS (RMS), which occurs in an estimated 80% to 85% of patients with the condition, followed by primary progressive MS (PPMS) in an estimated 10% to 15%.^4^,^5^ Approximately 50% of patients who initially presented with RMS ultimately transition to secondary progressive MS (SPMS) after 10 to 20 years.^4^,^5^,^11^,^12^ In recent years, clinically isolated syndrome (CIS), a first clinical episode suggestive of MS, was added to the established classifications as an additional MS phenotype. Patients diagnosed with CIS experience clinical symptoms and have objective clinical findings (for example, from neurological exam and/or MRI) that correspond to the anatomical location suggested by the neurological symptoms. CIS is diagnosed with the first episode of symptoms typical of MS that lasts longer than 24 hours; however, patients with CIS do not meet diagnostic criteria for RMS.^4^,^5^,^13^ Up to 80% of patients diagnosed with CIS who have abnormal MRI findings will develop MS in the future, compared with 20% of patients with CIS who have normal MRI findings.^4^,^5^

RMS is characterized by recurrent relapses, in which symptoms usually develop over hours to days, in the absence of fever or infection, lasting up to several weeks before either partially or completely improving.^4^,^5^,^11^,^14^ Individuals whose disease has transitioned to SPMS experience a progressive worsening over time that is not often associated with relapses.^4^,^5^,^11^ Patients with PPMS have slowly progressive symptoms from onset that may go unnoticed for some time.^5^,^11^,^12^

MS symptoms vary by location and severity of the lesions occurring within the CNS. The most common symptoms include optic neuritis, Lhermitte sign (an electric shocklike sensation occurring with neck flexion), sensory loss or altered sensations, motor weakness, fatigue, urinary dysfunction, cognitive dysfunction, incoordination, and walking difficulties.^4^,^12^ In RMS, visual, sensory, and brainstem relapses are often seen earlier in the disease, whereas pyramidal, bowel and/or bladder, and cerebellar relapses are often seen later in the disease course.^14^ Patients with PPMS most often present clinically with progressive myelopathy, although sensory ataxia, cerebellar ataxia, cognitive symptoms, and progressive visual loss have been reported as well.^5^,^11^,^12^

## DIAGNOSTIC WORKUP

Unfortunately, no single test diagnoses MS. Rather, the diagnostic process entails obtaining a detailed medical history, including a description of the signs and symptoms of previous and/or current episode(s); performing a thorough neurologic exam to localize the lesion(s) and help determine if the episode and exam findings are consistent with MS; and ordering appropriate diagnostic studies. Keeping the differential diagnosis in mind, providers should order lab testing to rule out conditions that may mimic MS.

To be diagnosed with MS, a patient must fulfill the 2017 McDonald criteria, which require the presence of two or more lesions developing over time (“dissemination in time”) in two or more locations within the CNS (“dissemination in space”). Presence of two or more lesions along with evidence of a new lesion in a different location in a person who has had one or more attacks is sufficient for diagnosis. If a patient does not meet these requirements, then the criteria also stipulate other scenarios involving these variables that allow for MS diagnosis. The presence of oligoclonal bands in the cerebrospinal fluid (CSF), but not in the serum, may indicate MS, for example.^13^ If a patient is suspected of having a progressive course and does not meet the dissemination in time criterion clinically or radiologically, then CSF analysis demonstrating presence of CSF-specific oligoclonal bands confirms the diagnosis.^4^,^5^,^12^,^13^ Brain MRI, which allows for visualization of lesions, is recommended for all patients suspected of having MS, and spinal MRI is recommended when clinically indicated.

## DISEASE MANAGEMENT

Since approval of the first DMT—interferon-beta-1b—in 1993, many more medications that lower relapse rates, reduce new disease activity, and delay disease progression have been approved by the FDA.^4^,^15^ After confirming the diagnosis of MS, one of the provider's main goals is to initiate the most appropriate DMT for the patient and follow up with periodic MRIs of the brain and spinal cord to evaluate for disease activity. This multifaceted decision is based on the patient's disease activity (disease phenotype, relapse rate, lesion load and location, degree of recovery from relapses, level of disability), patient preference, medication safety profile, and pregnancy plans, among other factors. With the continuously growing armamentarium of effective MS DMTs, the approach to selecting which treatment the patient should start with and/or switch to has been a hot topic.^15^,^16^

In recent years, the main MS treatment strategies endorsed among experts have been the traditional approach and the early aggressive approach. The traditional, or escalation, approach involves beginning with a low-/modest-efficacy DMT and switching to a higher efficacy DMT when breakthrough disease occurs as evidenced by a clinical relapse or new radiologic activity (for example, new or enhancing lesions). The early aggressive, or induction, approach entails bypassing the low-/modest-efficacy DMTs and starting with a high-efficacy DMT. Each approach has its rationales and challenges, but it is known that many of the currently approved high-efficacy DMTs have superior long-term clinical benefit to the less or modestly effective DMTs, both in and out of clinical trials.^15^,^16^

There is no set standard or algorithm for the management of MS. After discussing the available treatment options, patients and providers may agree to follow the escalation approach for several reasons, such as relapse rate, lesion load and location, level of disability, family planning, and concerns about the DMT safety profile or potential long-term adverse reactions. Alternatively, after a discussion with the provider, a patient with a higher risk tolerance may choose to start a more effective, aggressive treatment immediately for some of the same reasons or other considerations, including reduction of future disability and concern for poor relapse recovery. Growing evidence suggests that the early aggressive approach to MS management has been associated with more positive long-term outcomes, including reductions in disability accumulation and in risk of conversion from RMS to SPMS; however, more significant risks may accompany it.^15^,^16^ TREAT-MS and DELIVER-MS are two ongoing clinical trials that are exploring the risks and benefits of each strategy.

### Traditional DMTs

Currently, the DMTs that fall within the traditional category are glatiramer acetate, interferon-beta medications, fumarates, teriflunomide, and sphingosine 1-phosphate (S1P) receptor modulators (Table 1).

**TABLE 1.** Traditional DMTs^5^,^18^,^20^-^31^

DMT	Route	Select common and/or serious adverse reactions	Monitoring
Glatiramer acetate (Copaxone, generics)^a^	Subcutaneous	Injection site redness and swelling, postinjection reaction (flushing, palpitations, tachycardia, anxiety, breathing problems, throat constriction, and/or urticaria), vasodilation, rash, breathing problems, chest pain, and lipoatrophy	None
*Interferon-betas^a^:*		Injection site reactions, flulike symptoms, lymphopenia, transaminitis, depression, suicide, psychotic symptoms, seizures, anaphylaxis, congestive heart failure, thrombotic microangiopathy, pulmonary arterial hypertension, and autoimmune disorders	CBC w/diff and LFTs prior to initiation; at months 1, 3, and 6; and then every 6 months
Interferon-beta-1b (Betaseron, Extavia)	Subcutaneous		
Interferon-beta-1a (Rebif)	Subcutaneous		
Interferon-beta-1a (Avonex)	I.M.		
Peginterferon-beta-1a (Plegridy)	Subcutaneous, I.M.		
*Fumarates^a^:*	P.O.	Flushing, abdominal pain, diarrhea, and nausea	CBC w/diff and LFTs prior to initiation and then every 6 months
Dimethyl fumarate (Tecfidera, generics)			
Diroximel fumarate (Vumerity)			
Monomethyl fumarate (Bafiertam)			
Teriflunomide (Aubagio, generics)^a^	P.O.	Headache, nausea, diarrhea, alopecia, and elevated ALT. Cases of peripheral neuropathy have been reported.	Prior to initiation: CBC w/diff, LFTs, TB screening, and hepatitis panel
			During therapy: Monthly LFTs for first 6 months, then CBC w/diff and LFTs every 6 months
*S1P receptor modulators:*	P.O.	Upper respiratory infections, transaminitis, hypertension, UTI, back pain, and dizziness	Prior to initiation: CBC w/diff, LFTs, VZV-IgG, ECG, CYP2C9 genotyping, and ophthalmic exam
Fingolimod (Gilenya, Tascenso ODT, generics)^a^,^b^			During therapy: FDO required for fingolimod and for certain patients receiving other S1P receptor modulators; ophthalmic exam 3 to 4 months after first dose to rule out macular edema; CBC w/diff and LFTs every 6 months
Siponimod (Mayzent)^a^			
Ozanimod (Zeposia)^a^			
Ponesimod (Ponvory)^a^			

Abbreviations: ALT, alanine aminotransferase; CBC, complete blood cell count; CYP2C9, cytochrome P450 2C9; DMT, disease-modifying therapy; FDO, first-dose observation; IgG, immunoglobulin G; LFTs, liver function tests; ODT, orally disintegrating tablet; S1P, sphingosine 1-phosphate; TSH, thyroid-stimulating hormone; UTI, urinary tract infection; VZV, varicella zoster virus; w/diff, with differential.

a Indicated for the treatment of relapsing forms of MS, to include clinically isolated syndrome, relapsing-remitting disease, and active secondary progressive disease, in adults.

b Indicated for the treatment of relapsing forms of MS in children and adolescents 10 years of age or older.

Note: This is not a complete list of all prescribing and monitoring considerations. Providers should refer to appropriate guidelines and drug package inserts.

***Glatiramer acetate***. Glatiramer acetate, which is administered subcutaneously, works by shifting the immune response from a proinflammatory to an anti-inflammatory state.^17^,^18^ Ensuring patients inject their medication after allowing it to warm to room temperature and with proper technique typically helps reduce injection site adverse reactions, but applying a cold compress may also be beneficial. Rotating the injection sites and using proper technique are essential for preventing skin changes such as lipoatrophy. Postinjection reaction is possible, though it is typically transient and self-limited. Additionally, transient chest pain, either as part of a postinjection reaction or unaccompanied by any other symptoms, is possible. Consider further evaluation if symptoms deviate from what is usually reported. Glatiramer acetate is one of the preferred medications for women who are planning to conceive; although it is not recommended to continue the medication during pregnancy, it does not require a washout period (the period of time from drug discontinuation to elimination from the body), and accidental exposure in the first trimester has been reported as safe.^17^,^19^

***Interferon-beta***. Immune system effects of interferon-beta therapies are thought to include decreasing inflammation by enhancing suppressor T-cell activity and reducing proinflammatory cytokine production and antigen presentation.^17^ Injection site reactions and flulike symptoms are common.^20^-^23^ Adequate hydration, use of analgesics or antipyretics, and rotation of injection sites may reduce these adverse reactions. Thyroid dysfunction that is not well controlled should prompt the provider to consider a DMT that is not an interferon. Interferon-beta therapies are similarly preferred for individuals of childbearing potential, as a washout period is not required given the data on the relative safety of accidental first-trimester exposure, with the exception of a possible slightly increased risk of decreased infant birth weight.^17^,^19^

***Fumarates***. The fumarates include dimethyl fumarate, diroximel fumarate, and monomethyl fumarate (MMF). MMF, the active metabolite of dimethyl fumarate and diroximel fumarate, activates the nuclear factor (erythroid-derived 2)-like 2 (Nrf2) pathway, which increases antioxidant activity and is involved in the cellular response to the harmful effects of oxidative stress.^24^-^26^ All three DMTs require a lower starting dose followed by an increase to a maintenance dose to improve the tolerability of common adverse reactions.^24^-^26^ To help reduce gastrointestinal adverse reactions, patients using dimethyl fumarate should be advised to consider taking it with high-fat foods; patients using MMF should be advised to consider taking MMF without food, though this is not required.^25^,^26^ Patients should avoid taking diroximel fumarate with a high-fat, high-calorie meal, as it can affect absorption.^25^ Fumarates also carry risks of lymphopenia and liver injury. If lymphopenia (lymphocyte count of less than 500 cells/mcL) is present and persists for more than 6 months, treatment should be discontinued due to risk of opportunistic infections.^5^,^24^ Individuals planning to become pregnant should discontinue treatment, and fumarates should not be used during pregnancy.^9^,^17^,^19^

***Teriflunomide***. Teriflunomide works by inhibiting dihydroorotate dehydrogenase, an enzyme involved in *de novo* pyrimidine synthesis, thereby inhibiting the proliferation of rapidly dividing T and B cells and leaving resting lymphocytes unaltered.^27^ Teriflunomide has several important drug interactions, including with oral contraceptives, rosuvastatin, and warfarin, to name a few.^27^ The drug carries a boxed warning for hepatotoxicity. Cases of neutropenia, lymphocytopenia, and thrombocytopenia have been reported.^5^ Teriflunomide also has a boxed warning for embryofetal toxicity; therefore, it should be avoided in individuals of childbearing potential who are not using effective contraception and in pregnant individuals.^19^ Pregnancy should be excluded prior to initiation of the medication. Because teriflunomide can be detected in semen, men should use reliable contraceptive methods while on treatment. If planning to conceive, both men and women taking teriflunomide should undergo an accelerated elimination procedure with cholestyramine or activated charcoal after drug discontinuation, due to the long amount of time required for drug clearance from the body.^17^,^19^

***S1P receptor modulators***. The S1P receptor modulators include fingolimod, siponimod, ozanimod, and ponesimod. In 2010, fingolimod was the first S1P receptor modulator and first oral DMT to come to market to treat adults with RMS, and it was later approved to treat children 10 years of age or older with RMS.^28^ All four drugs work by binding to the S1P receptors of the lymphocytes in the periphery, preventing them from leaving the lymph nodes and reducing the number of lymphocytes in the peripheral blood that may cross into the CNS.^28^-^31^ Fingolimod binds to S1P receptors 1, 3, 4, and 5; siponimod and ozanimod bind to S1P receptors 1 and 5; and ponesimod only binds to S1P receptor 1.^28^-^31^

Initiation of fingolimod requires a first-dose observation (FDO) to monitor for signs and symptoms of bradycardia with hourly vital signs for 6 hours and pre- and postobservation ECGs for signs of postdose atrioventricular block.^28^ The other S1P receptor modulators are prescribed with an initial titration schedule to reduce first-dose adverse reactions and allow eligible patients to skip the FDO. Cases of progressive multifocal leukoencephalopathy (PML) have been reported in patients taking fingolimod, typically occurring after at least 2 years of treatment.^28^ Disease reactivation within 4 months of discontinuing an S1P receptor modulator is a known risk.^28^,^32^

S1P receptor modulators are associated with an increased risk of macular edema, typically within the first 4 months of treatment, as well as lymphopenia and liver transaminase elevations.^5^,^28^-^31^ Due to the risk of varicella zoster virus (VZV) reactivation with S1P receptor modulators, patients who do not have immunity to VZV must be vaccinated before starting treatment.^28^ Finally, for siponimod, cytochrome P450 2C9 (CYP2C9) genotyping is needed prior to initiation to identify individuals in whom the medication is contraindicated or who need a reduced dose.^28^-^31^

Pregnancy is typically protective against MS relapses, but it does not necessarily protect against the risk of disease reactivation associated with stopping fingolimod specifically.^19^ Due to the risk of fetal malformations with use of S1P receptor modulators, women planning to conceive should discontinue treatment, with close monitoring due to the risk of relapse.^17^,^19^,^28^ The washout period is 2 to 3 months for fingolimod, 10 days for siponimod, 3 months for ozanimod, and 7 days for ponesimod.^28^-^31^ The obstetric provider may consider early fetal ultrasound for major malformations if the patient becomes pregnant while on treatment.^17^,^19^,^28^

### Early aggressive DMTs

The next group of DMTs are considered early aggressive treatment options and include natalizumab, the anti-CD20 therapies, alemtuzumab, and cladribine (Table 2).

**TABLE 2.** Early aggressive DMTs^5^,^33^,^35^-^37^,^45^,^46^,^55^

DMT	Route	Select common and/or serious adverse reactions	Monitoring
Natalizumab (Tysabri, biosimilar available)^a^	I.V.	Headache, fatigue, arthralgia, UTI, lower respiratory tract infection, gastroenteritis, vaginitis, depression, pain in extremity, abdominal discomfort, diarrhea, and rash	Prior to initiation: Brain MRI, CBC w/diff, LFTs, and anti-JCV antibody with index
			During therapy: CBC w/diff and LFTs every 6 months and anti-JCV antibody with index every 3 to 6 months
*Anti-CD20 monoclonal antibodies:*		Upper and lower respiratory tract infections, skin infections, and infusion reactions (pruritus, rash, urticaria, erythema, bronchospasm, throat irritation, oropharyngeal pain, dyspnea, pharyngeal or laryngeal edema, flushing, hypotension, pyrexia, fatigue, headache, dizziness, nausea, tachycardia, and anaphylaxis)	Prior to initiation: Screening for hepatitis B virus, IGRA testing (TB screening), VZV-IgG testing, testing for serum immunoglobulins, LFTs, CBC w/diff, and lymphocyte subset panel (to obtain baseline CD19+ cell count)
Rituximab (Rituxan and biosimilars)∗	I.V.		During therapy: CBC w/diff, LFTs, and lymphocyte subset panel (CD19+) every 6 months (prior to infusion); MRI brain annually; IgG levels at least annually; standard cancer screenings
Ocrelizumab (Ocrevus)^a^,^b^	I.V.		
Ublituximab (Briumvi)^a^	I.V.		
Ofatumumab (Kesimpta)^a^	Subcutaneous	Upper respiratory tract infections, headache, systemic injection-related reactions, and local injection site reactions	
Alemtuzumab (Lemtrada)^c^	I.V.	Rash, headache, pyrexia, nasopharyngitis, nausea, vomiting, UTI, fatigue, insomnia, upper respiratory tract infection, herpes viral infection, urticaria, pruritus, thyroid gland disorders, fungal infection, arthralgia, extremity pain, back pain, diarrhea, sinusitis, oropharyngeal pain, paresthesia, dizziness, abdominal pain, flushing, and infusion reactions (anaphylaxis, angioedema, bronchospasm, hypotension, chest pain, bradycardia, tachycardia, hypertension, headache, pyrexia, rash, fatigue, dysgeusia, dyspepsia, dizziness, pain, urticaria, and nausea)	Prior to initiation: CBC w/diff, serum creatinine, urine protein-to-creatinine ratio, UA with urine cell counts, TSH, LFTs, VZV IgG, TB screening, and skin exam
			During therapy: CBC w/diff, serum creatinine levels, and UA with urine cell counts monthly; TSH every 3 months; LFTs periodically; and skin exam annually, all continuing until 48 months after last treatment course. Standard cancer screenings.
Cladribine (Mavenclad)^d^	P.O.	Upper respiratory tract infection, headache, lymphopenia, and infections (particularly herpes zoster)	Prior to initiation: Brain MRI; standard cancer screenings; pregnancy testing; CBC w/diff; LFTs; and HIV, hepatitis B and C, TB, and VZV-IgG testing
			During therapy: CBC w/diff before each treatment course and at months 2 and 6 after the start of each treatment course. If lymphocytes ≤200 cells/mcL, place patient on herpes prophylaxis and repeat labs monthly.

Abbreviations: CBC, complete blood cell count; DMT, disease-modifying therapy; IgG, immunoglobulin G; IGRA, interferon-gamma release assay; JCV, John Cunningham virus; LFTs, liver function tests; TB, tuberculosis; TSH, thyroid-stimulating hormone; UA, urinalysis; UTI, urinary tract infection; VZV, varicella zoster virus; w/diff, with differential.

∗ Off-label for treatment of MS.

a Indicated for the treatment of relapsing forms of MS, to include clinically isolated syndrome, relapsing-remitting disease, and active secondary progressive disease, in adults.

b Indicated for the treatment of primary progressive MS in adults.

c Indicated for the treatment of relapsing forms of MS, to include relapsing-remitting disease and active secondary progressive disease, in adults who have had an inadequate response to two or more drugs indicated for the treatment of MS. Not recommended for use in clinically isolated syndrome.

d Indicated for the treatment of relapsing forms of MS, to include relapsing-remitting disease and active secondary progressive disease, in adults who have had an inadequate response to, or are unable to tolerate, an alternative drug indicated for the treatment of MS. Not recommended for use in clinically isolated syndrome.

Note: This is not a complete list of all prescribing and monitoring considerations. Providers should refer to appropriate guidelines and drug package inserts.

***Natalizumab***. Natalizumab blocks alpha-4 integrin function by binding to the alpha-4 subunit on all leukocytes except neutrophils, resulting in the inability of these leukocytes to cross the blood-brain barrier into the CNS.^33^ Natalizumab, which is administered intravenously, carries a boxed warning for increased risk of PML, which may lead to death or significant disability; patient risk factors include presence of anti-John Cunningham virus (JCV) antibodies in serum or plasma, treatment duration of greater than 2 to 3 years, and prior immunosuppressant use.^33^,^34^ If the patient has antibodies to JCV, the provider may consider an alternative agent or may use natalizumab for a shorter duration before switching to a different DMT. The latter strategy has challenges, including the risk of disease reactivation following drug cessation. Patients may also decide to remain on natalizumab due to treatment response; however, this population requires more frequent imaging due to the risk of PML and, based on more recent evidence, extension of the typical infusion interval to reduce this risk.^34^

Natalizumab should be avoided during pregnancy. If the patient is planning to conceive, then their provider may opt to switch treatment. However, given the high risk of relapse with withdrawal, the specialist may consider continuing treatment throughout pregnancy with close monitoring by a dedicated team, including a high-risk obstetrician and a neonatologist, to evaluate for complications and possible hematologic abnormalities in the newborn.^17^,^19^

***Anti-CD20 monoclonal antibodies***. Anti-CD20 monoclonal antibodies have been widely used since their approval for MS. They selectively deplete CD20-expressing B cells through antibody-dependent cellular cytolysis and complement-mediated lysis.^35^-^37^ For some years before the first FDA-approved anti-CD20 therapy, providers used rituximab for PPMS to delay disease progression based on results from the OLYMPUS trial.^38^ Later came the ORATORIO clinical trial, which sought to investigate ocrelizumab versus placebo in younger patients with PPMS; it found that those treated with ocrelizumab had lower rates of clinical and MRI progression compared with those on placebo.^39^

In 2017, the FDA approved ocrelizumab, administered via I.V. infusions, for RMS or PPMS following the results of the ORATORIO (PPMS) and OPERA I and II (RMS) trials.^36^ Ofatumumab was approved in 2020 for RMS as the first and only anti-CD20 therapy for patient self-administration by subcutaneous injection.^35^ In 2022, the FDA approved ublituximab-xiiy for treatment of RMS via I.V. infusions. Ublituximab-xiiy is glycoengineered to exclude specific sugar molecules that may interfere with the binding of the Fc region of the drug to the Fc receptor expressed by natural killer cells, thereby enhancing affinity and resulting in more efficient B-cell depletion.^37^

All anti-CD20 therapies are contraindicated in patients with active hepatitis B infection. Cases of reactivation of viruses such as herpes zoster have been reported; therefore, VZV-IgG testing and vaccination may be considered before starting treatment.^16^ Once on treatment, an annual MRI of the brain with and without contrast should be completed for surveillance, as with other MS DMTs; however, the provider may order it sooner (for example, at 3 to 6 months) if the patient is switching from another DMT such as natalizumab, as cases of carryover and non-carryover PML have been confirmed after switching to ocrelizumab specifically.^16^,^36^ Additionally, labs should be drawn before each infusion to rule out active infection or significant lab abnormalities due to cases of neutropenia in patients receiving ocrelizumab.^15^,^40^

Anti-CD20 therapies are becoming a common treatment option for women of childbearing years, as they are highly effective in preventing inflammatory activity in the pre-pregnancy and postpartum phases, with effects lasting up to 18 months after the last infusion.^19^,^41^-^44^ Counseling points for family planning discussions should include the timing of pregnancy and infusions as well as available data regarding outcomes of exposed neonates. Women on ocrelizumab and ublituximab-xiiy may be advised to wait 6 months from the last infusion before attempting to conceive, per the prescribing information, or 3 months from the last infusion, based on data showing that elimination of the drugs occurs within 3 to 6 months after exposure and that minimal transfer across the placenta occurs during the first trimester; regardless, patients should not attempt to conceive while on the drugs.^17^,^19^,^36^,^37^,^41^,^42^ Anti-CD20 therapies should then continue to be held while individuals are attempting to conceive and during pregnancy. Those on ofatumumab may be advised to continue the medication to reduce the risk of relapse until they are ready to conceive and then to stop the medication at that time, although the prescribing information advises waiting 6 months after discontinuing the medication to attempt to conceive.^17^,^19^,^35^,^41^

***Alemtuzumab***. Alemtuzumab's proposed mechanism of action involves selectively binding to surface antigen CD52, which is highly expressed on T and B lymphocytes, resulting in antibody-dependent cellular cytolysis and complement-mediated lysis.^15^,^16^,^45^ The drug was approved by the FDA in 2014 for the treatment of RMS via I.V. infusions.^45^ Herpes prophylaxis is recommended for 2 months postinfusion or until the patient's CD4 lymphocyte count is 200 cells/mcL or greater, whichever happens later.^45^ Alemtuzumab is contraindicated in patients with HIV due to its prolonged reduction of CD4 lymphocyte counts.^45^ Due to the risks of autoimmunity, infusion reactions, stroke, and malignancies, alemtuzumab carries a boxed warning. More specifically, the drug may result in autoantibody formation, increasing the risk of serious autoimmune-mediated conditions such as thyroid disorders, immune thrombocytopenia, glomerular nephropathies, autoimmune hemolytic anemia, and autoimmune pancytopenia, and it may increase the risk of malignancies such as thyroid cancer, melanoma, lymphoproliferative disorders, and lymphoma.^45^

Alemtuzumab causes cytokine release syndrome, resulting in infusion reactions that can be serious and life-threatening.^45^ Patients should be premedicated with corticosteroids such as I.V. methylprednisolone, and in some cases also with oral or I.V. antihistamines and oral antipyretics, to reduce the severity of infusion reactions. Cases of placental transfer of antithyroid antibodies resulting in neonatal Graves disease have been reported.^17^,^19^,^41^,^45^ Although the FDA recommends waiting at least 4 months postinfusion for women to conceive, some experts recommend that women postpone pregnancy for at least 12 months after the last infusion (the time at which the development of autoimmune diseases associated with alemtuzumab peaks), since recommended treatments for those diseases may be contraindicated during pregnancy.^19^,^45^

***Cladribine***. Cladribine is a prodrug that enters lymphocytes in an inactive form and must be activated by a specific phosphorylating enzyme found in high levels in T and B lymphocytes. Once activated, the drug interferes with DNA synthesis and repair of the T and B lymphocytes, resulting in lymphocyte depletion through cell death.^46^ Treatment is administered over 2 courses (years) during cycles (months) 1 and 2 of each course.^46^ Contraindications include hepatitis B, tuberculosis, HIV infection, current malignancy, pregnancy, and breastfeeding during the treatment period.^16^,^46^

In addition to other baseline testing, VZV-IgG testing can help to identify those who may benefit from vaccination before starting treatment.^5^,^46^ Patients should be placed on herpes prophylaxis and should complete monthly lab testing if their lymphocyte count is greater than or equal to 200 cells/mcL.^46^ The drug package insert contains information on when to hold treatment based on lymphocyte count. Due to increased risk of malignancy, the treatment is limited to 2 years.^46^ Both men and women must use effective contraception to prevent pregnancy during dosing of the drug and for at least 6 months after the last dose of each treatment course due to risk of fetal harm.^17^,^41^,^46^

## PRACTICE IMPLICATIONS

### Multidisciplinary team approach

Caring for a patient with MS requires a multidisciplinary care team led by neurology with support from primary care, ophthalmology, urology, gastroenterology, psychiatry, psychology, and physical therapy. PCPs play a vital role in ensuring optimal outcomes for patients with MS who, in addition to facing this complex disease, are not immune to other conditions (for example, hypertension, diabetes, or cardiac disease). In addition to completing the annual physical, PCPs should remind patients to complete applicable standard cancer screenings, which are crucial given the risk of malignancy associated with some DMTs. Providers should encourage patients to make healthy lifestyle modifications such as eating a healthy diet, exercising, and quitting smoking if applicable. It is also critical to have open family planning discussions to help to identify those who need contraception, immunizations, or specialist consultation.

### Immunizations

An attempt should be made to administer any missing vaccines before starting DMT. This is especially true ahead of initiating S1P receptor modulator therapy, for which VZV vaccination is required in those who are antibody-negative at least 4 weeks before starting treatment.^28^ To reduce wait time between vaccination and treatment, a single dose rather than two doses of measles-mumps-rubella (MMR) and VZV live attenuated vaccines (followed by immunogenicity testing) might be sufficient for protection.^47^ However, live attenuated vaccines must otherwise be avoided in patients on DMT due to immunocompromise.^48^

In general, non-live (inactivated) vaccines are acceptable for people with MS, but recommendations on the timing of these vaccines vary based on DMT.^41^,^48^,^49^ Non-live vaccines may be administered anytime for patients on glatiramer acetate or interferon-betas with adequate vaccine response.^48^-^50^ It is possible that some patients on teriflunomide, dimethyl fumarate, or natalizumab may have reduced response to non-live vaccinations.^48^-^50^ The S1P receptor modulators and anti-CD20 therapies are associated with reduced vaccine response.^48^-^50^

Based on the VELOCE clinical trial results, patients on ocrelizumab may benefit from timing their non-live (inactivated) vaccines between 12 weeks postinfusion and 4 to 6 weeks before their next infusion.^48^,^51^ According to one study, patients on natalizumab, dimethyl fumarate, diroximel fumarate, and interferon-beta demonstrated preserved humoral response following COVID-19 vaccination but those treated with ocrelizumab demonstrated a blunted response.^52^ Patients on alemtuzumab have reduced vaccine responses if vaccinated within 6 months postinfusion, a better response if they wait at least 6 months postinfusion, and a full response if they are vaccinated once reaching complete immune reconstitution.^53^ A small number of patients treated with cladribine in the MAGNIFY-MS trial received VZV and influenza vaccines, and they developed protection irrespective of lymphocyte count, likely due to the incomplete depletion and quick recovery of immature B cells.^54^

## CONCLUSION

MS is a chronic demyelinating disease of the CNS, management of which is growing in complexity as new treatment options emerge. Collaboration between MS specialists and PCPs in caring for patients with MS is essential in providing comprehensive, well-coordinated care. PCPs promote patient adherence to treatment, identify clinically significant lab abnormalities, manage comorbid conditions, treat infections, and follow standard cancer screening and immunization recommendations. Staying informed about what is new and current in the MS treatment landscape can help the provider engage in the patient's treatment plan and deliver individualized care.
